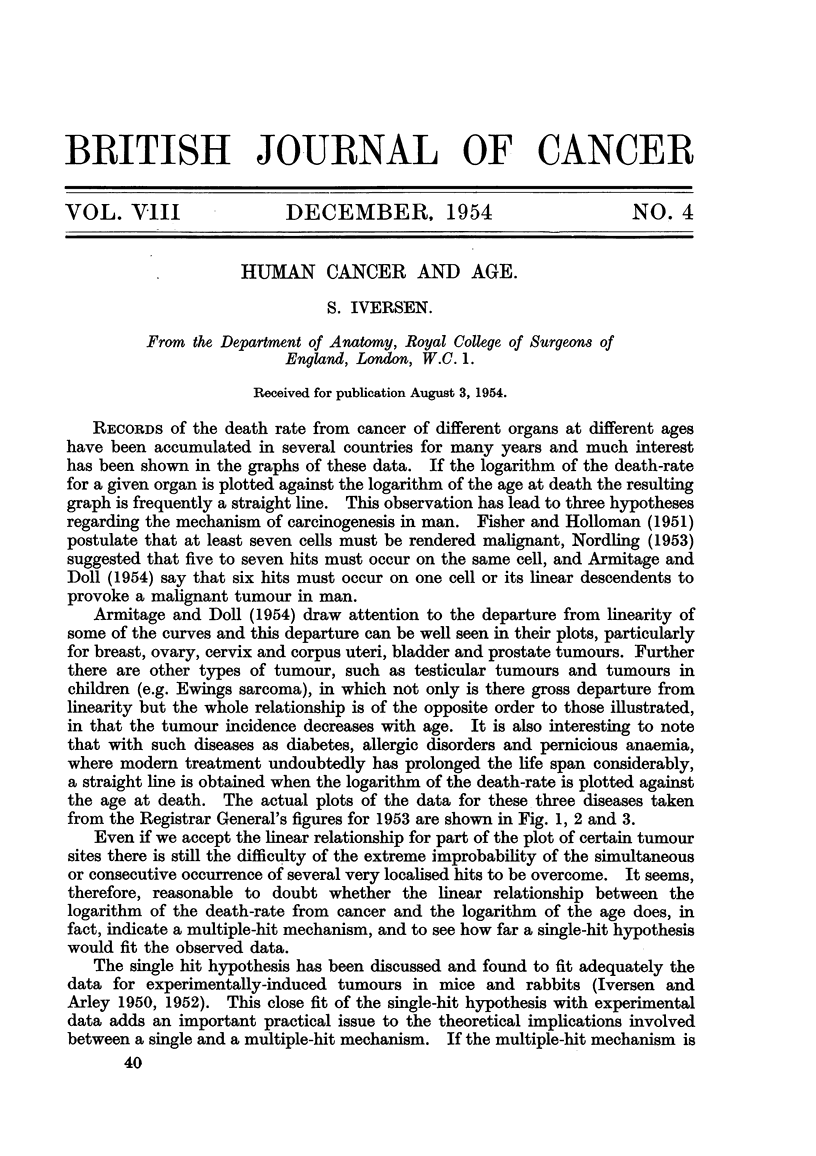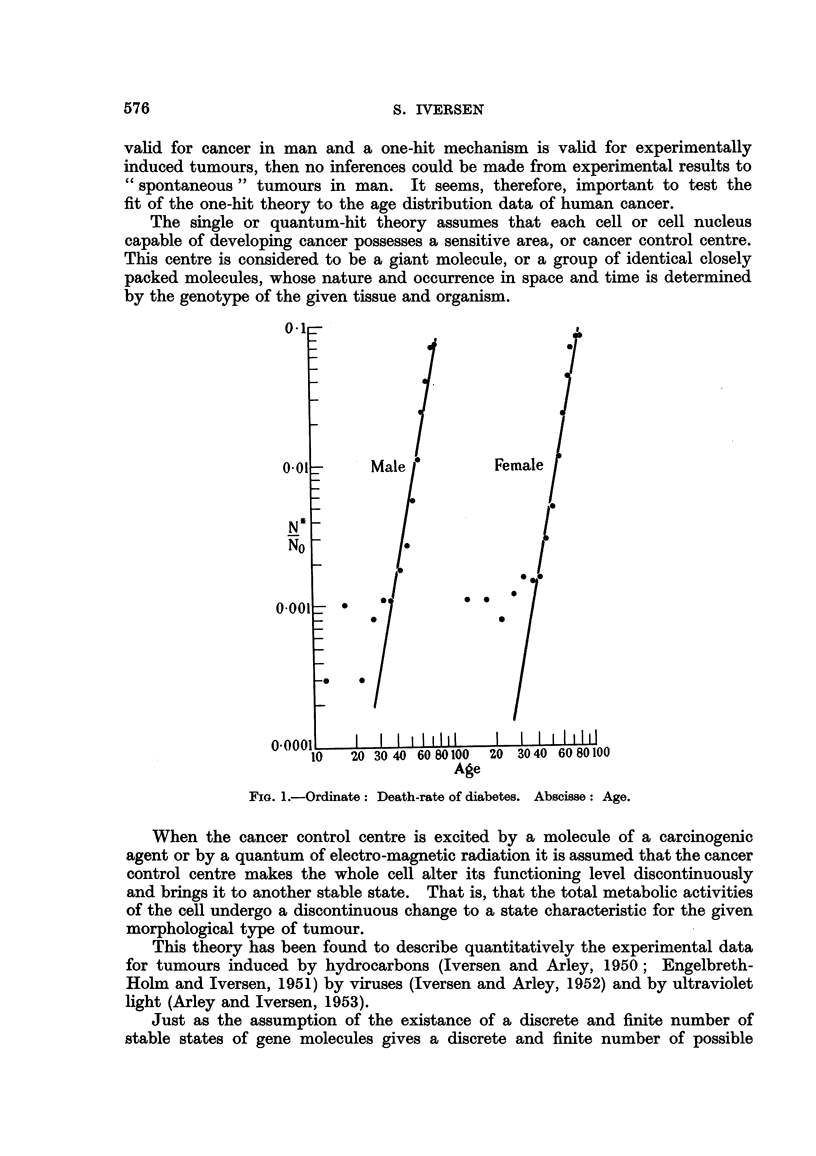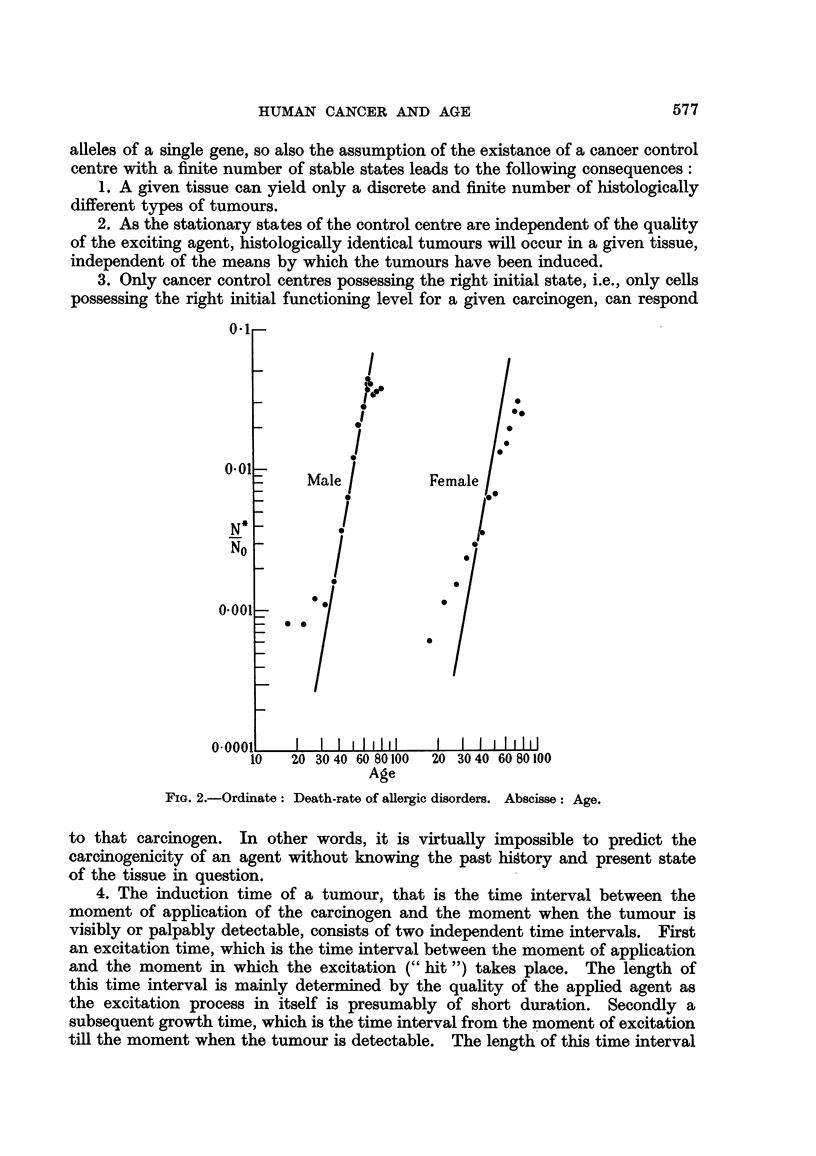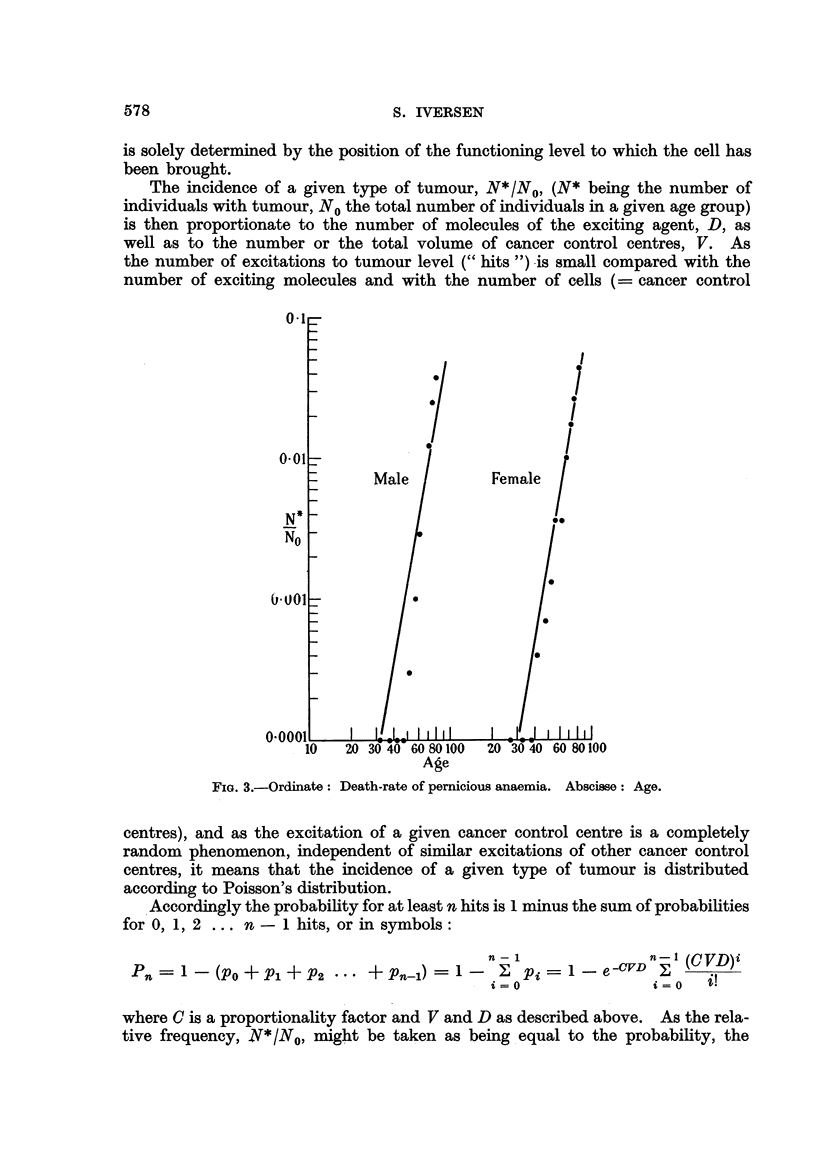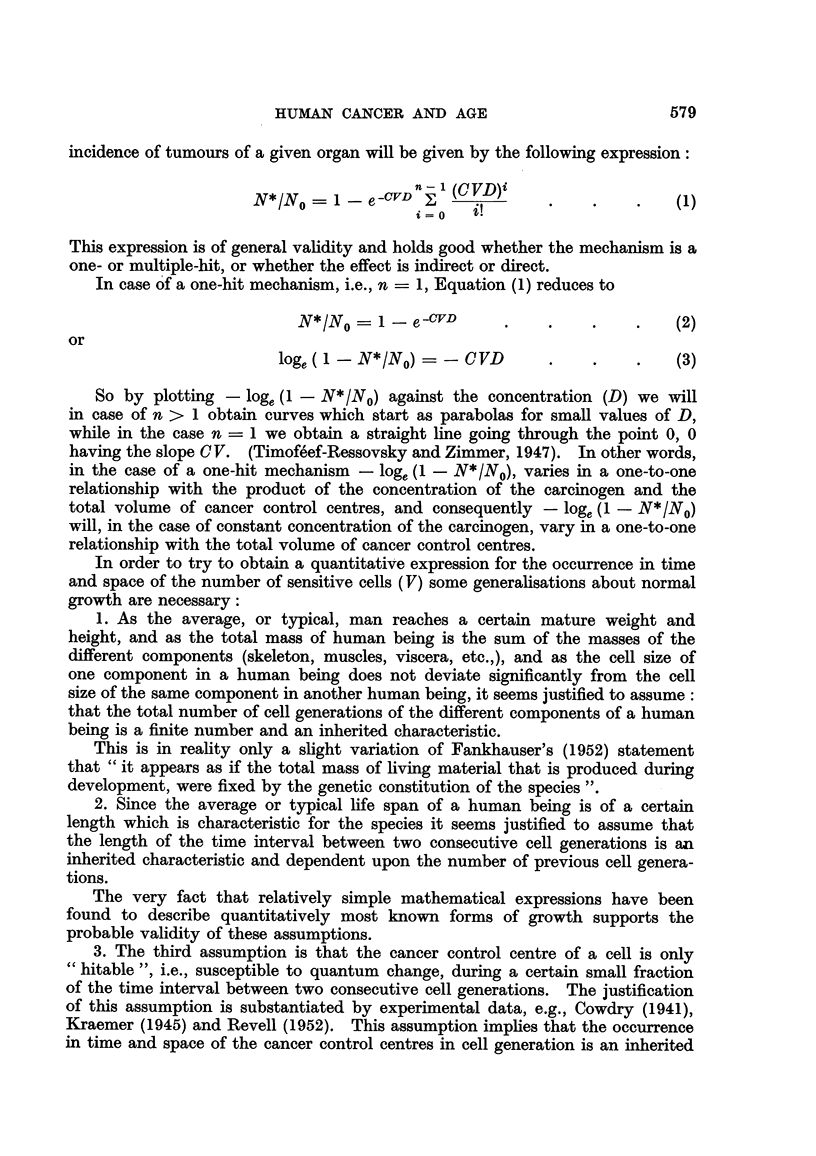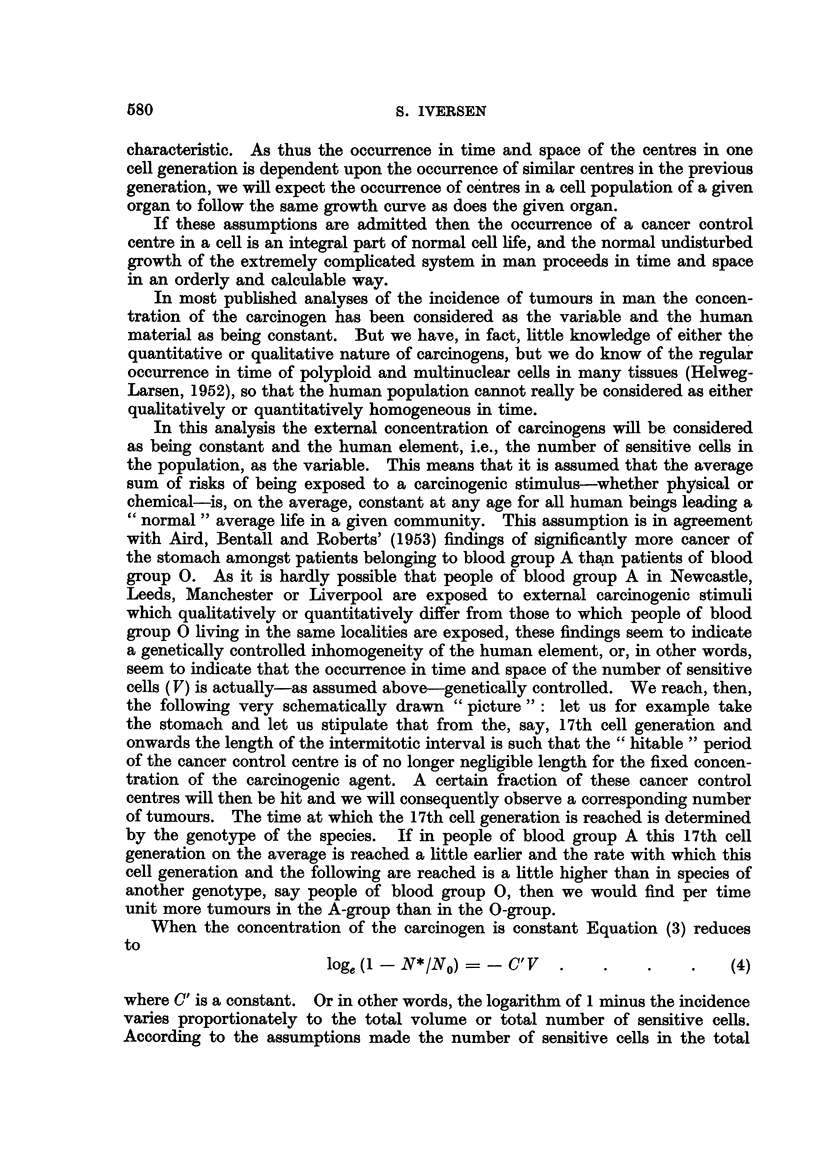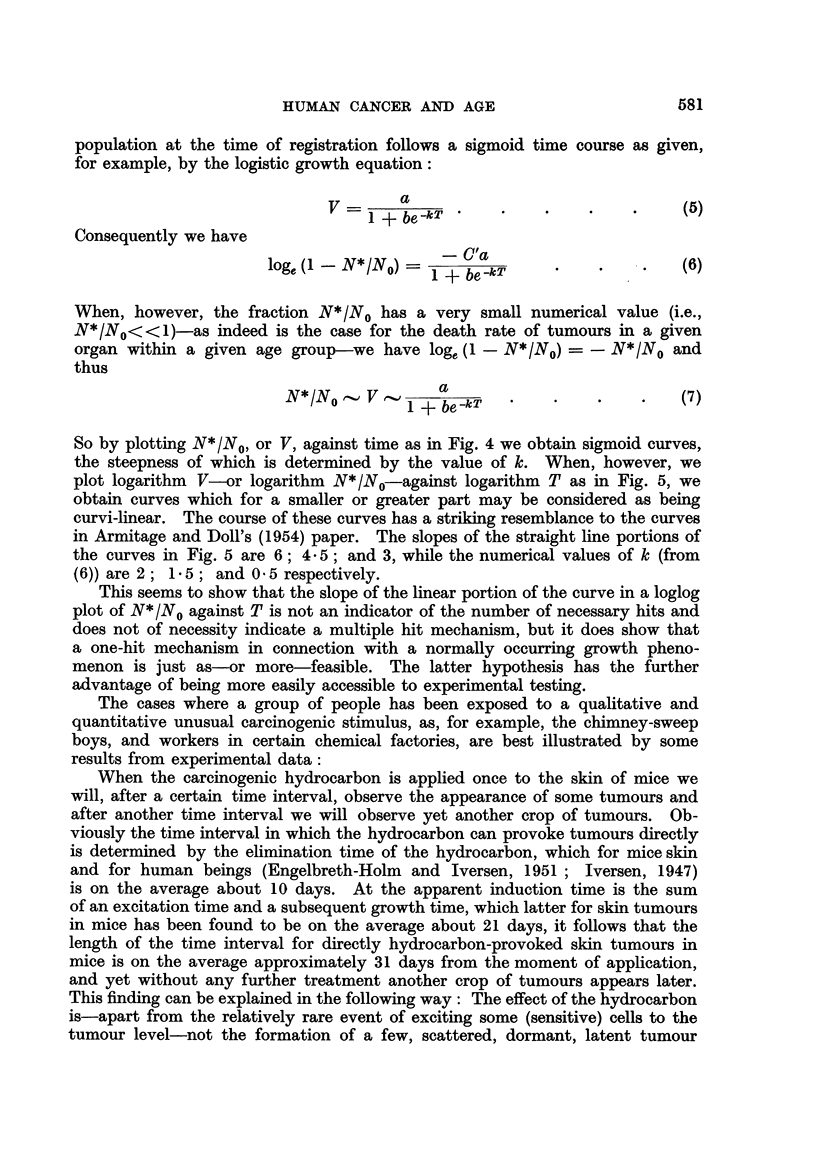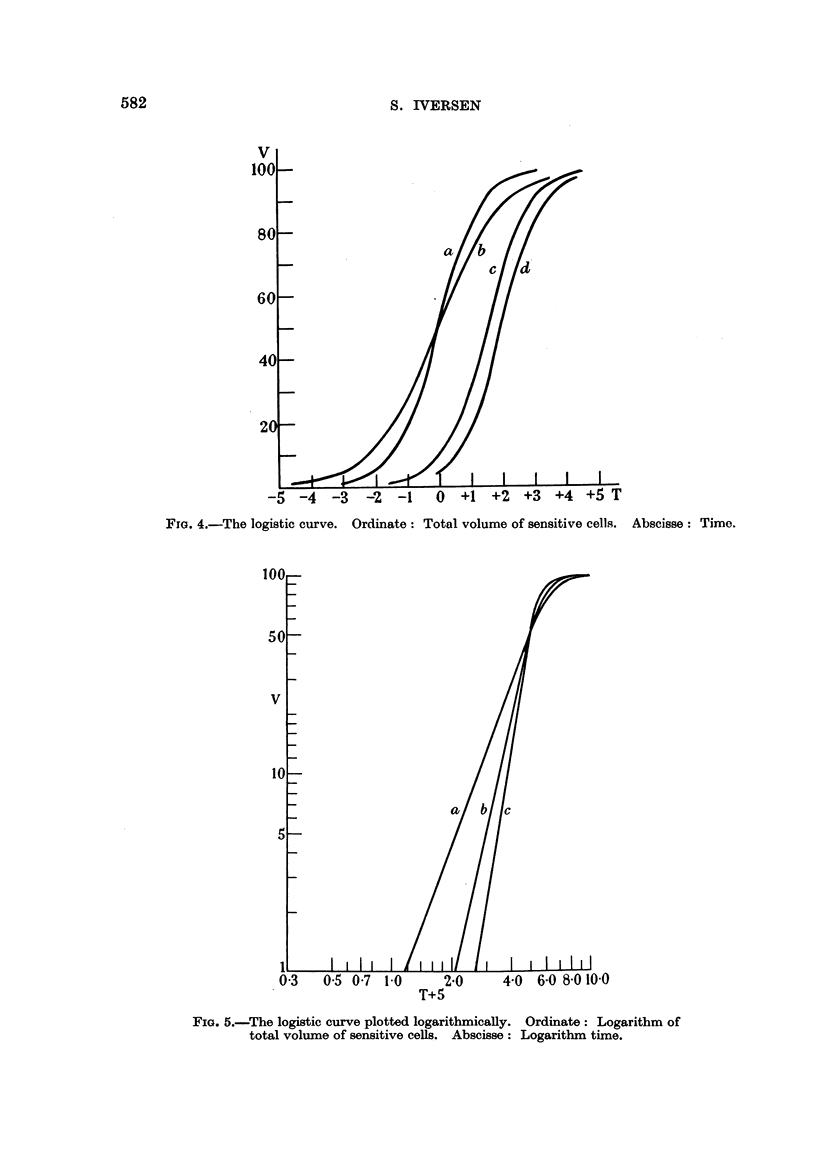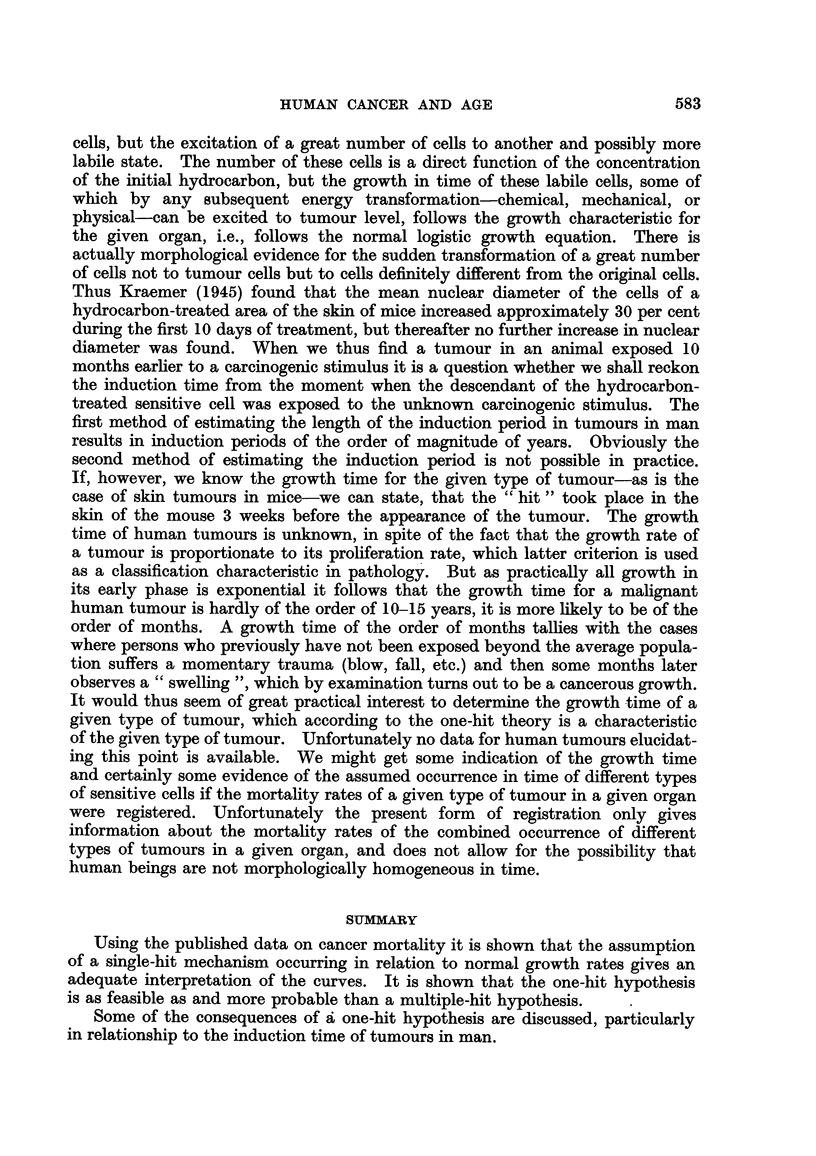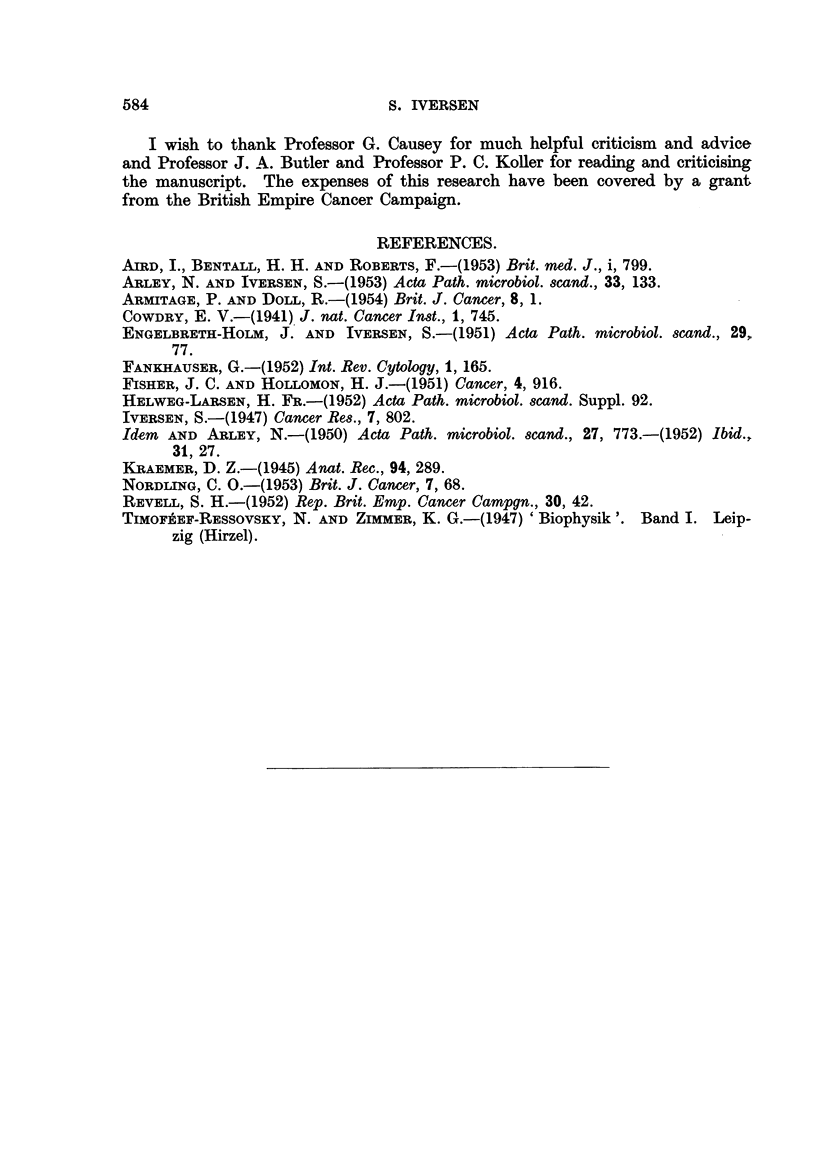# Human Cancer and Age

**DOI:** 10.1038/bjc.1954.62

**Published:** 1954-12

**Authors:** S. Iversen


VOL. VIII DECEMBER, 1954 NO. 4

### HUMAN CANCER AND AGE.

S. IVERSEN.

*From the Department of Anatomy, Royal College of Surgeons of England, London, W.C.* 1.

Received for publication August 3, 1954.

RECORDS of the death rate from cancer of different organs at different ages have been accumulated in several countries for many years and much interest has been shown in the graphs of these data. If the logarithm of the death-rate for a given organ is plotted against the logarithm of the age at death the resulting graph is frequently a straight line. This observation has lead to three hypotheses regarding the mechanism of carcinogenesis in man. Fisher and Holloman (1951) postulate that at least seven cells must be rendered malignant, Nordling (1953) suggested that five to seven hits must occur on the same cell, and Armitage and Doll (1954) say that six hits must occur on one cell or its linear descendents to provoke a malignant tumour in man.

Armitage and Doll (1954) draw attention to the departure from linearity of some of the curves and this departure can be well seen in their plots, particularly for breast, ovary, cervix and corpus uteri, bladder and prostate tumours. Further there are other types of tumour, such as testicular tumours and tumours in children (e.g. Ewings sarcoma), in which not only is there gross departure from linearity but the whole relationship is of the opposite order to those illustrated, in that the tumour incidence decreases with age. It is also interesting to note that with such diseases as diabetes, allergic disorders and pernicious anaemia, where modern treatment undoubtedly has prolonged the life span considerably, a straight line is obtained when the logarithm of the death-rate is plotted against the age at death. The actual plots of the data for these three diseases taken from the Registrar General's figures for 1953 are shown in Fig. 1, 2 and 3.

Even if we accept the linear relationship for part of the plot of certain tumour sites there is still the difficulty of the extreme improbability of the simultaneous or consecutive occurrence of several very localised hits to be overcome. It seems, therefore, reasonable to doubt whether the linear relationship between the logarithm of the death-rate from cancer and the logarithm of the age does, in fact, indicate a multiple-hit mechanism, and to see how far a single-hit hypothesis would fit the observed data.

The single hit hypothesis has been discussed and found to fit adequately the data for experimentally-induced tumours in mice and rabbits (Iversen and Arley 1950, 1952). This close fit of the single-hit hypothesis with experimental data adds an important practical issue to the theoretical implications involved between a single and a multiple-hit mechanism. If the multiple-hit mechanism is valid for cancer in man and a one-hit mechanism is valid for experimentally induced tumours, then no inferences could be made from experimental results to "spontaneous" tumours in man. It seems, therefore, important to test the fit of the one-hit theory to the age distribution data of human cancer.

The single or quantum-hit theory assumes that each cell or cell nucleus capable of developing cancer possesses a sensitive area, or cancer control centre. This centre is considered to be a giant molecule, or a group of identical closely packed molecules, whose nature and occurrence in space and time is determined by the genotype of the given tissue and organism.

FIG. 1.—Ordinate : Death-rate of diabetes. Abscisse : Age.

When the cancer control centre is excited by a molecule of a carcinogenic agent or by a quantum of electro-magnetic radiation it is assumed that the cancer control centre makes the whole cell alter its functioning level discontinuously and brings it to another stable state. That is, that the total metabolic activities of the cell undergo a discontinuous change to a state characteristic for the given morphological type of tumour.

This theory has been found to describe quantitatively the experimental data for tumours induced by hydrocarbons (Iversen and Arley, 1950; Engelbreth-Holm and Iversen, 1951) by viruses (Iversen and Arley, 1952) and by ultraviolet light (Arley and Iversen, 1953).

Just as the assumption of the existance of a discrete and finite number of stable states of gene molecules gives a discrete and finite number of possible alleles of a single gene, so also the assumption of the existance of a cancer control centre with a finite number of stable states leads to the following consequences :

1. A given tissue can yield only a discrete and finite number of histologically different types of tumours.

2. As the stationary states of the control centre are independent of the quality of the exciting agent, histologically identical tumours will occur in a given tissue, independent of the means by which the tumours have been induced.

3. Only cancer control centres possessing the right initial state, i.e., only cells possessing the right initial functioning level for a given carcinogen, can respond

FIG. 2.—Ordinate : Death-rate of allergic disorders. Abscisse : Age.

to that carcinogen. In other words, it is virtually impossible to predict the carcinogenicity of an agent without knowing the past history and present state of the tissue in question.

4. The induction time of a tumour, that is the time interval between the moment of application of the carcinogen and the moment when the tumour is visibly or palpably detectable, consists of two independent time intervals. First an excitation time, which is the time interval between the moment of application and the moment in which the excitation (" hit ") takes place. The length of this time interval is mainly determined by the quality of the applied agent as the excitation process in itself is presumably of short duration. Secondly a subsequent growth time, which is the time interval from the moment of excitation till the moment when the tumour is detectable. The length of this time interval is solely determined by the position of the functioning level to which the cell has been brought.

The incidence of a given type of tumour, $N^*/N_0$, ($N^*$ being the number of individuals with tumour, $N_0$ the total number of individuals in a given age group) is then proportionate to the number of molecules of the exciting agent, $D$, as well as to the number or the total volume of cancer control centres, $V$. As the number of excitations to tumour level (" hits ") is small compared with the number of exciting molecules and with the number of cells (= cancer control

FIG. 3.—Ordinate : Death-rate of pernicious anaemia. Abscisse : Age.

centres), and as the excitation of a given cancer control centre is a completely random phenomenon, independent of similar excitations of other cancer control centres, it means that the incidence of a given type of tumour is distributed according to Poisson's distribution.

Accordingly the probability for at least $n$ hits is 1 minus the sum of probabilities for 0, 1, 2 ... $n - 1$ hits, or in symbols :

$$P_n = 1 - (p_0 + p_1 + p_2 \ldots + p_{n-1}) = 1 - \sum_{i=0}^{n-1} p_i = 1 - e^{-CVD} \sum_{i=0}^{n-1} \frac{(CVD)^i}{i!}$$

where $C$ is a proportionality factor and $V$ and $D$ as described above. As the relative frequency, $N^*/N_0$, might be taken as being equal to the probability, the incidence of tumours of a given organ will be given by the following expression :

$$N^*/N_0 = 1 - e^{-CVD} \sum_{i=0}^{n-1} \frac{(CVD)^i}{i!} \quad . \quad . \quad . \quad (1)$$

This expression is of general validity and holds good whether the mechanism is a one- or multiple-hit, or whether the effect is indirect or direct.

In case of a one-hit mechanism, i.e., $n = 1$, Equation (1) reduces to

$$N^*/N_0 = 1 - e^{-CVD} \quad . \quad . \quad . \quad . \quad (2)$$

or

$$\log_e ( 1 - N^*/N_0) = - CVD \quad . \quad . \quad . \quad (3)$$

So by plotting $- \log_e (1 - N^*/N_0)$ against the concentration ($D$) we will in case of $n > 1$ obtain curves which start as parabolas for small values of $D$, while in the case $n = 1$ we obtain a straight line going through the point 0, 0 having the slope $CV$. (Timoféef-Ressovsky and Zimmer, 1947). In other words, in the case of a one-hit mechanism $- \log_e (1 - N^*/N_0)$, varies in a one-to-one relationship with the product of the concentration of the carcinogen and the total volume of cancer control centres, and consequently $- \log_e (1 - N^*/N_0)$ will, in the case of constant concentration of the carcinogen, vary in a one-to-one relationship with the total volume of cancer control centres.

In order to try to obtain a quantitative expression for the occurrence in time and space of the number of sensitive cells ($V$) some generalisations about normal growth are necessary :

1. As the average, or typical, man reaches a certain mature weight and height, and as the total mass of human being is the sum of the masses of the different components (skeleton, muscles, viscera, etc.,), and as the cell size of one component in a human being does not deviate significantly from the cell size of the same component in another human being, it seems justified to assume : that the total number of cell generations of the different components of a human being is a finite number and an inherited characteristic.

This is in reality only a slight variation of Fankhauser's (1952) statement that " it appears as if the total mass of living material that is produced during development, were fixed by the genetic constitution of the species ".

2. Since the average or typical life span of a human being is of a certain length which is characteristic for the species it seems justified to assume that the length of the time interval between two consecutive cell generations is an inherited characteristic and dependent upon the number of previous cell generations.

The very fact that relatively simple mathematical expressions have been found to describe quantitatively most known forms of growth supports the probable validity of these assumptions.

3. The third assumption is that the cancer control centre of a cell is only " hitable ", i.e., susceptible to quantum change, during a certain small fraction of the time interval between two consecutive cell generations. The justification of this assumption is substantiated by experimental data, e.g., Cowdry (1941), Kraemer (1945) and Revell (1952). This assumption implies that the occurrence in time and space of the cancer control centres in cell generation is an inherited characteristic. As thus the occurrence in time and space of the centres in one cell generation is dependent upon the occurrence of similar centres in the previous generation, we will expect the occurrence of centres in a cell population of a given organ to follow the same growth curve as does the given organ.

If these assumptions are admitted then the occurrence of a cancer control centre in a cell is an integral part of normal cell life, and the normal undisturbed growth of the extremely complicated system in man proceeds in time and space in an orderly and calculable way.

In most published analyses of the incidence of tumours in man the concentration of the carcinogen has been considered as the variable and the human material as being constant. But we have, in fact, little knowledge of either the quantitative or qualitative nature of carcinogens, but we do know of the regular occurrence in time of polyploid and multinuclear cells in many tissues (Helweg-Larsen, 1952), so that the human population cannot really be considered as either qualitatively or quantitatively homogeneous in time.

In this analysis the external concentration of carcinogens will be considered as being constant and the human element, i.e., the number of sensitive cells in the population, as the variable. This means that it is assumed that the average sum of risks of being exposed to a carcinogenic stimulus—whether physical or chemical—is, on the average, constant at any age for all human beings leading a " normal " average life in a given community. This assumption is in agreement with Aird, Bentall and Roberts' (1953) findings of significantly more cancer of the stomach amongst patients belonging to blood group A than patients of blood group O. As it is hardly possible that people of blood group A in Newcastle, Leeds, Manchester or Liverpool are exposed to external carcinogenic stimuli which qualitatively or quantitatively differ from those to which people of blood group O living in the same localities are exposed, these findings seem to indicate a genetically controlled inhomogeneity of the human element, or, in other words, seem to indicate that the occurrence in time and space of the number of sensitive cells ($V$) is actually—as assumed above—genetically controlled. We reach, then, the following very schematically drawn " picture ": let us for example take the stomach and let us stipulate that from the, say, 17th cell generation and onwards the length of the intermitotic interval is such that the " hitable " period of the cancer control centre is of no longer negligible length for the fixed concentration of the carcinogenic agent. A certain fraction of these cancer control centres will then be hit and we will consequently observe a corresponding number of tumours. The time at which the 17th cell generation is reached is determined by the genotype of the species. If in people of blood group A this 17th cell generation on the average is reached a little earlier and the rate with which this cell generation and the following are reached is a little higher than in species of another genotype, say people of blood group O, then we would find per time unit more tumours in the A-group than in the O-group.

When the concentration of the carcinogen is constant Equation (3) reduces to

$$\log_e (1 - N^*/N_0) = - C'V \quad . \quad . \quad . \quad . \quad (4)$$

where $C'$ is a constant. Or in other words, the logarithm of 1 minus the incidence varies proportionately to the total volume or total number of sensitive cells. According to the assumptions made the number of sensitive cells in the total population at the time of registration follows a sigmoid time course as given, for example, by the logistic growth equation :

$$V = \frac{a}{1 + be^{-kT}} \quad . \quad . \quad . \quad . \quad . \quad . \qquad (5)$$

Consequently we have

$$\log_e (1 - N^*/N_0) = \frac{- C'a}{1 + be^{-kT}} \quad . \quad . \quad . \quad . \qquad (6)$$

When, however, the fraction $N^*/N_0$ has a very small numerical value (i.e., $N^*/N_0 << 1$)—as indeed is the case for the death rate of tumours in a given organ within a given age group—we have $\log_e (1 - N^*/N_0) = - N^*/N_0$ and thus

$$N^*/N_0 \sim V \sim \frac{a}{1 + be^{-kT}} \quad . \quad . \quad . \quad . \quad . \qquad (7)$$

So by plotting $N^*/N_0$, or $V$, against time as in Fig. 4 we obtain sigmoid curves, the steepness of which is determined by the value of $k$. When, however, we plot logarithm $V$—or logarithm $N^*/N_0$—against logarithm $T$ as in Fig. 5, we obtain curves which for a smaller or greater part may be considered as being curvi-linear. The course of these curves has a striking resemblance to the curves in Armitage and Doll's (1954) paper. The slopes of the straight line portions of the curves in Fig. 5 are 6 ; 4·5 ; and 3, while the numerical values of $k$ (from (6)) are 2 ; 1·5 ; and 0·5 respectively.

This seems to show that the slope of the linear portion of the curve in a loglog plot of $N^*/N_0$ against $T$ is not an indicator of the number of necessary hits and does not of necessity indicate a multiple hit mechanism, but it does show that a one-hit mechanism in connection with a normally occurring growth phenomenon is just as—or more—feasible. The latter hypothesis has the further advantage of being more easily accessible to experimental testing.

The cases where a group of people has been exposed to a qualitative and quantitative unusual carcinogenic stimulus, as, for example, the chimney-sweep boys, and workers in certain chemical factories, are best illustrated by some results from experimental data :

When the carcinogenic hydrocarbon is applied once to the skin of mice we will, after a certain time interval, observe the appearance of some tumours and after another time interval we will observe yet another crop of tumours. Obviously the time interval in which the hydrocarbon can provoke tumours directly is determined by the elimination time of the hydrocarbon, which for mice skin and for human beings (Engelbreth-Holm and Iversen, 1951 ; Iversen, 1947) is on the average about 10 days. At the apparent induction time is the sum of an excitation time and a subsequent growth time, which latter for skin tumours in mice has been found to be on the average about 21 days, it follows that the length of the time interval for directly hydrocarbon-provoked skin tumours in mice is on the average approximately 31 days from the moment of application, and yet without any further treatment another crop of tumours appears later. This finding can be explained in the following way : The effect of the hydrocarbon is—apart from the relatively rare event of exciting some (sensitive) cells to the tumour level—not the formation of a few, scattered, dormant, latent tumour

FIG. 4.—The logistic curve. Ordinate : Total volume of sensitive cells. Abscisse : Time.

FIG. 5.—The logistic curve plotted logarithmically. Ordinate : Logarithm of total volume of sensitive cells. Abscisse : Logarithm time.

cells, but the excitation of a great number of cells to another and possibly more labile state. The number of these cells is a direct function of the concentration of the initial hydrocarbon, but the growth in time of these labile cells, some of which by any subsequent energy transformation—chemical, mechanical, or physical—can be excited to tumour level, follows the growth characteristic for the given organ, i.e., follows the normal logistic growth equation. There is actually morphological evidence for the sudden transformation of a great number of cells not to tumour cells but to cells definitely different from the original cells. Thus Kraemer (1945) found that the mean nuclear diameter of the cells of a hydrocarbon-treated area of the skin of mice increased approximately 30 per cent during the first 10 days of treatment, but thereafter no further increase in nuclear diameter was found. When we thus find a tumour in an animal exposed 10 months earlier to a carcinogenic stimulus it is a question whether we shall reckon the induction time from the moment when the descendant of the hydrocarbon-treated sensitive cell was exposed to the unknown carcinogenic stimulus. The first method of estimating the length of the induction period in tumours in man results in induction periods of the order of magnitude of years. Obviously the second method of estimating the induction period is not possible in practice. If, however, we know the growth time for the given type of tumour—as is the case of skin tumours in mice—we can state, that the " hit " took place in the skin of the mouse 3 weeks before the appearance of the tumour. The growth time of human tumours is unknown, in spite of the fact that the growth rate of a tumour is proportionate to its proliferation rate, which latter criterion is used as a classification characteristic in pathology. But as practically all growth in its early phase is exponential it follows that the growth time for a malignant human tumour is hardly of the order of 10–15 years, it is more likely to be of the order of months. A growth time of the order of months tallies with the cases where persons who previously have not been exposed beyond the average population suffers a momentary trauma (blow, fall, etc.) and then some months later observes a " swelling ", which by examination turns out to be a cancerous growth. It would thus seem of great practical interest to determine the growth time of a given type of tumour, which according to the one-hit theory is a characteristic of the given type of tumour. Unfortunately no data for human tumours elucidating this point is available. We might get some indication of the growth time and certainly some evidence of the assumed occurrence in time of different types of sensitive cells if the mortality rates of a given type of tumour in a given organ were registered. Unfortunately the present form of registration only gives information about the mortality rates of the combined occurrence of different types of tumours in a given organ, and does not allow for the possibility that human beings are not morphologically homogeneous in time.

### SUMMARY

Using the published data on cancer mortality it is shown that the assumption of a single-hit mechanism occurring in relation to normal growth rates gives an adequate interpretation of the curves. It is shown that the one-hit hypothesis is as feasible as and more probable than a multiple-hit hypothesis.

Some of the consequences of a one-hit hypothesis are discussed, particularly in relationship to the induction time of tumours in man.

I wish to thank Professor G. Causey for much helpful criticism and advice and Professor J. A. Butler and Professor P. C. Koller for reading and criticising the manuscript. The expenses of this research have been covered by a grant from the British Empire Cancer Campaign.

### REFERENCES.

AIRD, I., BENTALL, H. H. AND ROBERTS, F.—(1953) *Brit. med. J.*, i, 799.

ARLEY, N. AND IVERSEN, S.—(1953) *Acta Path. microbiol. scand.*, **33**, 133.

ARMITAGE, P. AND DOLL, R.—(1954) *Brit. J. Cancer*, **8**, 1.

COWDRY, E. V.—(1941) *J. nat. Cancer Inst.*, **1**, 745.

ENGELBRETH-HOLM, J. AND IVERSEN, S.—(1951) *Acta Path. microbiol. scand.*, **29**, 77.

FANKHAUSER, G.—(1952) *Int. Rev. Cytology*, **1**, 165.

FISHER, J. C. AND HOLLOMON, H. J.—(1951) *Cancer*, **4**, 916.

HELWEG-LARSEN, H. FR.—(1952) *Acta Path. microbiol. scand.* Suppl. 92.

IVERSEN, S.—(1947) *Cancer Res.*, **7**, 802.

*Idem* AND ARLEY, N.—(1950) *Acta Path. microbiol. scand.*, **27**, 773.—(1952) *Ibid.*, **31**, 27.

KRAEMER, D. Z.—(1945) *Anat. Rec.*, **94**, 289.

NORDLING, C. O.—(1953) *Brit. J. Cancer*, **7**, 68.

REVELL, S. H.—(1952) *Rep. Brit. Emp. Cancer Campgn.*, **30**, 42.

TIMOFÉEF-RESSOVSKY, N. AND ZIMMER, K. G.—(1947) ‘ Biophysik ’. Band I. Leipzig (Hirzel).